# Transcriptomic Response in *Pseudomonas aeruginosa* towards Treatment with a Kaempferol Isolated from *Melastoma malabathricum* Linn Leaves

**DOI:** 10.1155/2020/6915483

**Published:** 2020-02-05

**Authors:** Mourouge Saadi Alwash, Wan Syaidatul Aqma, Wan Yaacob Ahmad, Nazlina Ibrahim

**Affiliations:** ^1^Department of Biology, College of Science, University of Babylon, Hillah, Iraq; ^2^School of Biosciences and Biotechnology, Faculty of Science and Technology, Universiti Kebangsaan Malaysia, 43600 Bangi, Selangor, Malaysia; ^3^School of Chemical Sciences and Food Technology, Faculty of Science and Technology, Universiti Kebangsaan Malaysia, 43600 Bangi, Selangor, Malaysia

## Abstract

*Pseudomonas aeruginosa* is one of the main causes of nosocomial infections and is frequently associated with opportunistic infections among hospitalized patients. Kaempferol-3-*O*-(2′,6′-di-*O-trans*-*p-*coumaroyl)-*β*-D glucopyranoside (*K*_F_) is an antipseudomonal compound isolated from the leaves of the native medicinal plant *Melastoma malabathricum*. Herein, an RNA-seq transcriptomic approach was employed to study the effect of *K*_F_ treatment on *P. aeruginosa* and to elucidate the molecular mechanisms underlying the response to *K*_F_ at two time points (6 h and 24 h incubation). Quantitative real-time PCR (qRT-PCR) was performed for four genes (*uvrD*, *sodM*, *fumC1*, and *rpsL*) to assess the reliability of the RNA-seq results. The RNA-seq transcriptomic analysis revealed that *K*_F_ increases the expression of genes involved in the electron transport chain (NADH-I), resulting in the induction of ATP synthesis. Furthermore, *K*_F_ also increased the expression of genes associated with ATP-binding cassette transporters, flagella, type III secretion system proteins, and DNA replication and repair, which may further influence nutrient uptake, motility, and growth. The results also revealed that *K*_F_ decreased the expression of a broad range of virulence factors associated with LPS biosynthesis, iron homeostasis, cytotoxic pigment pyocyanin production, and motility and adhesion that are representative of an acute *P. aeruginosa* infection profile. In addition, *P. aeruginosa* pathways for amino acid synthesis and membrane lipid composition were modified to adapt to *K*_F_ treatment. Overall, the present research provides a detailed view of *P. aeruginosa* adaptation and behaviour in response to *K*_F_ and highlights the possible therapeutic approach of using plants to combat *P. aeruginosa* infections.

## 1. Introduction

*Pseudomonas aeruginosa* sp. is deemed one of the major etiological agents of both acute and chronic human infections ranging from minor skin infections to persistent and often life-threatening diseases in hospitalized or immunocompromised patients [1, 2]. Infections caused by this organism are difficult to treat due to the ability of this bacterium to resist multiple classes of antibiotics [3]. Strains of *P. aeruginosa* are well known to employ their high levels of intrinsic and acquired resistance mechanisms to combat most antibiotics [4]. In addition, pathogenesis of *P. aeruginosa* is multifactorial, and many virulence factors are produced that include secreted factors such as cytotoxic pigment pyocyanin, siderophores, alkaline protease, elastase, exotoxin A, rhamnolipid structural component lipopolysaccharide, pili, flagella, and biofilm formation [5]. Therefore, alternative drugs and new therapeutic strategies that present novel avenues against *P. aeruginosa* infections are increasingly required and gaining more and more attention [4]. Previous studies by our research group demonstrated that *K*_F_ can induce *P. aeruginosa* cell wall damage [6, 7]. Thus, we decided to investigate the gene expression profile of *P. aeruginosa* growing in kaempferol-3-*O*-(2′,6′-di-*O-trans*-*p-*coumaroyl)-*β*-D glucopyranoside isolated from *Melastoma malabathricum* known to locals in Malaysia as “senduduk.” Next-generation sequencing (NGS) technology may provide a detailed view of *P. aeruginosa* adaptation and behaviour in response to *K*_F_ and could help researchers further understand the transcriptomic response of *P. aeruginosa* to *K*_F_ exposure [8]. We compared the transcriptional responses of *P. aeruginosa* upon exposure to *K*_F_ at an early time point (6 h incubation) and at a late time point (24 h incubation) to provide information about the *K*_F_ mechanism of action. Transcriptomic data highlighted a marked modulation of gene expression characterized by the induction of the expression of several genes involved in pathogenesis, iron acquisition, DNA replication and repair, and metabolic adaptation to *K*_F_ growth conditions. The results presented in this study provide a detailed view of gene expression changes in *P. aeruginosa* in response to *K*_F_ exposure, facilitating the understanding of the cellular strategies that are utilized under *K*_F_ exposure conditions and identifying a potential mechanism for the inhibition of *P. aeruginosa* after *K*_F_ exposure.

## 2. Materials and Methods

### 2.1. Bacteria and Growth Conditions

*P seudomonas aeruginosa* strain ATCC 10145 was cultivated in Nutrient Broth (Oxoid, UK) with a shaking incubator at 151 rpm for 3 to 6 h at 37°C to achieve log phase growth. At the log phase (∼6 h incubation), *K*_F_ was added to the *P. aeruginosa* culture in Mueller Hinton Broth (Oxoid, UK) at a density of 4 × 10^5^ CFU/mL to achieve a final concentration of 0.5 mg/mL dissolved in 5% dimethyl sulfoxide (DMSO). DMSO (5%) was used as a negative control for untreated cells. The cultures were incubated at 37°C with a shaking incubator at 200 rpm.

### 2.2. RNA Extraction, cDNA Library Construction, and Illumina Sequencing

Total RNA was extracted from *P. aeruginosa* (treated or untreated) and harvested after 6 h and 24 h of incubation. RNA was extracted using an innuPREP RNA Mini Kit (Analytik Jena Biometra, Germany). The quantity and integrity were first determined using a NanoDrop 2000 spectrophotometer (Thermo Fisher Scientific, USA) and Agilent 2100 Bioanalyzer (Agilent Technologies, Palo Alto, CA, USA). The total RNA was depleted of rRNA using a ScriptSeq™ Complete Kit (Bacteria; Epicenter, San Diego, CA, USA). Total RNA samples were used for cDNA synthesis. Magnetic beads with attached poly T oligos were used to purify mRNA from the total RNA. The mRNA was then cleaved into small fragments by the addition of RNA fragmentation solution. First strand cDNA was synthesized using random hexamer adaptors and StarScript Reverse Transcriptase, followed by the synthesis of second strand cDNA using ScriptSeq v2 Terminal Tagging Premix and DNA polymerase. Exonuclease and polymerase were used to blunt and adenylate the 3′ ends of the DNA fragments, and Illumina PE adapter oligonucleotides were ligated to prepare for hybridization. The cDNA fragments (280 bp) were purified using the Pure AMXP system (Beckman Coulter, Beverly, CA, USA). The cDNA fragments with ligated adaptor molecules were enriched using Illumina PCR Primer Cocktail in a 15-cycle PCR. Finally, the cDNA library was sequenced on the Illumina MiSeq platform (San Diego, CA, USA) using single-end technology in a single run at the Institute of Biosciences, Universiti Putra Malaysia. The Illumina MiSeq software was used to perform the original image processing for sequencing, base calling, and quality value calculations, where 50 bp single-end reads were obtained.

### 2.3. Analysis of the Differentially Expressed Genes (DEGs)

The raw reads were filtered to obtain the high-quality clean data by removing adaptor sequences and low-quality reads with the Phred quality score ≤30. The clean reads were then mapped to the *P. aeruginosa* PA01 genome (NCBI reference sequence, NC_002516.2; GenBank accession number AE004091.2). FASTQ read values were calculated and normalized to transform into expression values by using CLC Genomics Workbench version 6.5. Differential expression analysis (fold changes) for RNAseq data was performed to compare two different samples (untreated versus treated samples) using Kal's *Z*-test. Genes with average fold changes >2 and adjusted *p* values less than 0.05 (i.e., false discovery rate less than 5%) were identified as significant DEGs. To better understand the biological functions and the metabolic pathways of the identified genes, the DEGs were functionally classified due to Gene Ontology (GO) and Kyoto Encyclopedia of Genes and Genomes (KEGG) databases. The significant DEGs at both 6 h and 24 h were compiled and used to generate a Venn diagram through an online interactive tool [9]. The gene lists of unique and shared genes in each group identified in the Venn diagram were analysed using the Database for Annotation, Visualization, and Integrated Discovery (DAVID) (http://david.abcc.ncifcrf.gov/home.jsp). The DAVID database provides a comprehensive set of functional annotation tools to understand the biological meaning behind the DEGs, including visualizing genes on KEGG pathway maps. In addition, the obtained data were then compiled with public datasets downloaded from the *Pseudomonas* Genome Database (http://www.pseudomonas.com) for further analysis. The raw RNA-seq data have been submitted to the NCBI Sequence Read Archive (NCBI SRA) under GenBank accession no. SRP060687 (NCBI SRA, http://www.ncbi.nlm.nih.gov/sra?term=SRP060687).

### 2.4. Validation of DEGs by Quantitative Real-Time PCR (qRT-PCR)

In order to validate the RNA-seq data and to have a concise view of *P. aeruginosa* gene expression profiles over time, qRT-PCR was employed and gene expression levels were analysed on a subset of genes whose functions were documented to contribute to *P. aeruginosa* virulence. Four genes with different expression patterns at two time points were chosen for the validation of the RNA-seq results. The template cDNAs were synthesized from 1 *μ*g of total RNA using oligo (dT)_18_, random hexamer primers, and reverse transcriptase enzyme mix (Maxima First Strand cDNA Synthesis Kit; Thermo Scientific, USA). A Luminaris Color HiGreen Fluorescein qPCR Master Mix (Thermo Scientific, USA) was used as a labelling agent, and L-aspartate oxidase (*nadB*) served as an internal reference gene. The reaction mixture (20 *μ*L) contained 2 × Master Mix (10 *μ*L), 10 *μ*M forward and reverse primers (1.2 *μ*L and 0.6 *μ*L of each), template cDNA (2 *μ*L), and RNase-free water (6.8 *μ*L). The PCR program was as follows: 2 minutes (min) at 50°C, 10 min at 95°C, followed by 40 cycles for 15 seconds (sec) at 95°C, 40 cycles of 30 sec at 53°C and 40 cycles of 30 sec at 72°C. The reaction was performed on an iCycler iQ5 instrument (Bio-Rad Laboratories, Inc., Hercules, Canada). Two independent biological replicates were included for each sample. The relative expression of a target gene in comparison to a reference gene expression level was calculated using Relative Expression Software Tool Multiple Condition Solver REST-MCS©-version 2 (http://rest.gene-quantification.info).

## 3. Results

### 3.1. Transcriptomic Analysis

Genome-wide transcriptomic analysis was conducted to elucidate the mechanism through which *K*_F_ exerts its killing effect on *P. aeruginosa* using NGS technology. After statistical analysis (Kal's *Z*-test), 2405 of the 5681 genes that comprise the *P. aeruginosa* genome were found to be significantly differentially regulated (*p* ≤ 0.05). A total of 2405 differentially expressed genes were classified based on the *Pseudomonas* Genome Database, KEGG pathways, individual operons, and genes potentially encoding targets associated with virulence factors.

**Figure 1:**
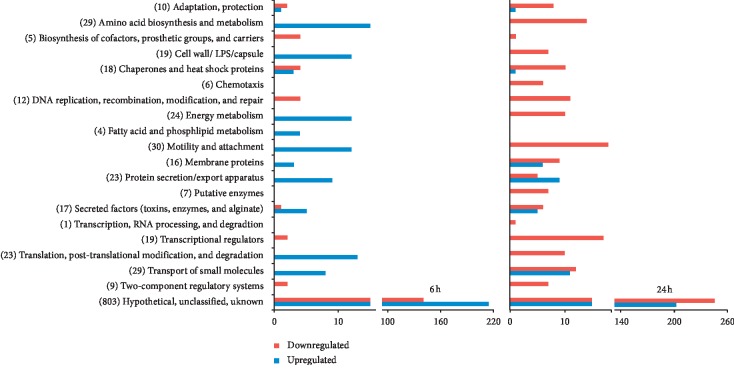
Histograms representing the number of genes based on their functional classes for *P. aeruginosa* and for the upregulated expression (blue bars) and downregulated expression (red bars) genes among the 1031 significantly expressed genes at both 6 h and 24 h exposure to *K*_F_ (0.5 mg/mL). The numbers in parentheses indicate the total number of genes for each functional class in both groups.

Further analysis revealed that 1031 genes showed statistically significant upregulation (>2.0-fold) or downregulation (>2.0-fold) of expression at 6 h and 24 h of exposure to *K*_F_.  Figure 1 illustrates that more downregulated genes in the functional classes were generally observed at 24 h compared to 6 h of incubation. The most noticeable number of downregulated genes among all functional classes was hypothetical, unclassified, and unknown (HUU) with unknown function.

**Figure 2:**
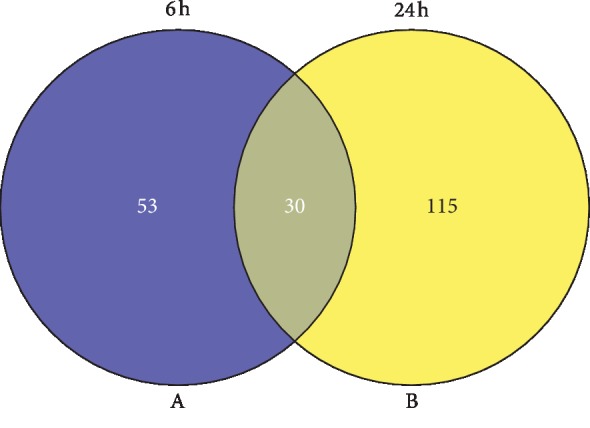
Venn diagram showing the overlap of significantly upregulated expression of genes at 6 h and 24 h exposure to *K*_F_ (0.5 mg/mL). (a) Venn diagram for early time point (6 h). (b) Venn diagram for late time point (24 h).

**Figure 3:**
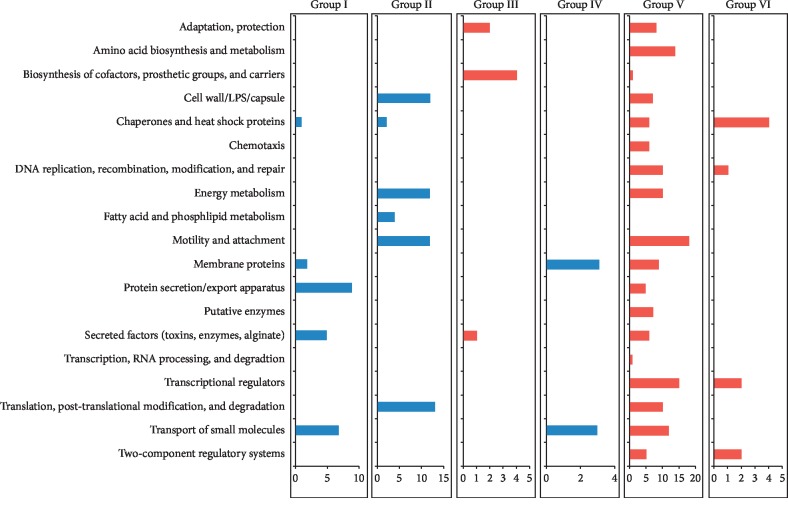
Classification of differentially upregulated and downregulated (total of 228) genes into six groups based on their functional classes at 6 h and 24 h exposure to *K*_F_ (0.5 mg/mL). Group I consisted of genes with upregulated expression at 6 h and 24 h. Group II consisted of genes with upregulated expression at 6 h without significant changes at 24 h. Group III consisted of genes with downregulated expression at 6 h without significant changes at 24 h. Group IV consisted of genes with upregulated expression at 24 h without significant changes at 6 h. Group V consisted of genes with downregulated expression at 24 h without significant changes at 6 h. Group VI consisted of genes with downregulated expression at 6 h and 24 h.

Note that 803 of 1031 genes were excluded as hypothetical proteins (HUUs). The Venn diagram for the remaining 228 genes at the two time points shows more uniquely over-represented genes at 24 h (115) than at 6 h (53), suggesting a difference between the early and late responses of *P. aeruginosa* to *K*_F_ (Figure 2). A total of 228 genes were placed in six groups based on their expression change direction (Figure 3).

#### 3.1.1. Group I: Genes with Upregulated Expression at 6 h and 24 h

Group I consisted of genes with upregulated expression at both 6 h and 24 h of exposure to *K*_F_ (Table 1). Growth under *K*_F_ exposure conditions induced changes in the expression of genes associated with ATP-binding cassette (ABC) transporters (*agtABCD* operon, PA4500), carbohydrate transporters (PA3190), and inorganic ion transporters (PA3514).

Group I also contained genes coding for the type III secretion system (T3SS). These genes with upregulated expression include those involved in the secretion and translocation machinery into the host cell plasma (*popBD* and *pcrV*); transcription and initiation (*exsCED*); chaperones that bind secreted proteins to facilitate the secretion process (*spcS*, *pcrH*, *pscG*, and *exsC*); and effector proteins that are injected into host cells (*exoSTY*; Table 1).

#### 3.1.2. Group II: Genes with Upregulated Expression at 6 h

Group II is composed of genes with expression levels that increased only at 6 h of exposure to *K*_F_ (Table 2). The expression of genes involved in the biosynthesis of several amino acids, including histidine (*hisC1* and *hisE*), arginine (*argF* and *argJ*), isoleucine (*ilvA1*), leucine (*leuA* and *leuC*), and phenylalanine (*pheA*), was increased after *K*_F_ exposure. In addition, we observed the overexpression of genes related to translation class, including genes encoding 30S and 50S ribosomal proteins (the two most upregulated genes, 30S and 50S, are listed in Table 2); aminoacyl-tRNA synthetases associated with tryptophan (*trpS*), tyrosine (*tyrZ*), glycine (*glyQ*), glutamine (*glnE*), valine (*valS*), proline (*pros*), cysteine (*cysS*), and isoleucine (*ileS*); translation initiation factor (*infC*); elongation factor G (*fusA2*); and peptide chain release factor (*prfC*) in response to *K*_F_ treatment.

As shown in Table 2, the upregulation of the expression of genes involved in the first step of long-chain fatty acid biosynthesis was also observed. The genes with upregulated expression include those encoding biotin carboxyl carrier protein (*accB*) and acetyl CoA carboxylase beta subunit (*accD*). In prokaryotes, this step involves the ATP-dependent carboxylation of acetyl coenzyme A (CoA) to form malonyl CoA by the enzyme acetyl CoA carboxylase. In addition, the expression of *fabA* and *fabB* genes, which are involved in the biosynthesis of unsaturated fatty acids (UFAs), was increased in *K*_F_ samples. Under anaerobic conditions, *P. aeruginosa* can utilize nitrate, nitrite, or nitrous oxide instead of oxygen as a terminal electron acceptor in the denitrification process. The expression of the nitric oxide reductase gene (*norB*) required for denitrification was upregulated. Furthermore, the most obvious upregulation of gene expression was found in the oxidative phosphorylation pathway. NADH created by the Krebs cycle can be fed into the oxidative phosphorylation pathway. The expression of NADH dehydrogenase I chain (*nuoBDFGHIJLMN)* in the oxidative phosphorylation pathway was increased. The *anr* gene encodes the transcriptional regulator Anr, which is involved in controlling *P. aeruginosa* gene expression under anaerobic conditions was significantly increased in *K*_F_-treated samples, with log2-fold changes of 3.84. Table 2 also shows that the expression of genes related to the flagella assembly pathway (*flgBCDEGIJK* and *fliEFG*) was also increased after *K*_F_ exposure.

#### 3.1.3. Group III: Genes with Downregulated Expression at 6 h

The expression of genes associated with adaptation, protection, and secreted factor functional class was downregulated (Table 3). The genes with downregulated expression include those associated with pyocin S2 (*pys2*) and pyocin S2 immunity protein (*imm2*). The expression of *cobODUJ* genes, which are involved in the aerobic cobalamin biosynthesis process (a cofactor for numerous enzymes mediating methylation, reduction, and intramolecular rearrangements), was reduced.

#### 3.1.4. Group IV: Genes with Upregulated Expression at 24 h

Exposure to *K*_F_ increased changes in the expression of genes associated with tripartite ATP-independent periplasmic transporters, including *dctP* (a C_4_ dicarboxylate-binding protein) and *dctQ* and *dctM* (C_4_ dicarboxylate transporters) (Table 4). *Pseudomonas. aeruginosa* preferentially uses C_4_ dicarboxylates, such as malate, fumarate, and succinate, as carbon and energy sources under anaerobic conditions.

#### 3.1.5. Group V: Genes with Downregulated Expression at 24 h

Group V is composed of genes with downregulated expression at 24 h of exposure to *K*_F_ (Table 5). The expression of the *pchR* gene, which encodes elements involved in iron Fe^3+^ acquisition, was reduced. In addition, growth under *K*_F_ exposure conditions reduced the changes in the expression of genes including members of the extracytoplasmic factor (ECF) subfamily (PA0471-PA0472, PA1300-1301, PA3899-PA3900, PA4895-PA4896, PA0149, PA1912, and PA2896). The expression of the *tonB* gene (TonB-dependent siderophore receptor) required for chelating Fe^3+^ was reduced. Furthermore, the expression of genes encoding fumarate hydratase (*fumC1*), superoxide dismutase (*sodM*), haemeoxygenase (*hemO*), and oxidoreductase (PA0853 and PA3768) was downregulated.

Table 5 also shows that the expression of virulence-associated genes that are involved in phenazine-1-carboxylic acid (PCA) biosynthesis (*phzA1B1C1A2B2*) and the conversion of PCA to pyocyanin (*phzMS*) were decreased. The expression of several genes associated with Sec system proteins was significantly altered. Exposure to *K*_F_ reduced the changes in the expression of genes such as the inner membrane translocase subunit proteins (*secD*), a cytoplasmic membrane-associated ATPase (*secA*), and a chaperone (*secB*) that binds to presecretory target proteins. The results also showed a downregulation of the expression of the *mexGHI*-*opmD* efflux pump system in the *K*_F_-treated samples (Table 5). In addition, the expression of several genes involved in the LPS biosynthesis process, including *lpxA*, *lpxB*, *waaF*, *waaG*, *waaP*, PA4998, PA5007, PA5008, and *rmlA,* was decreased. Transcription data of *P. aeruginosa* showed a downregulation in the expression of type VI pili composed of *pilDFMNOPQUVWXY1*. The expression levels of *vfr* (virulence factor regulator) and *pilGHIJ-chpAB* (Chp chemosensory system) genes were significantly decreased according to the Log2-fold changes. The virulence-associated *fliC* gene, which encodes flagellin type B, was downregulated under *K*_F_ exposure conditions.

Growth under *K*_F_ conditions reduced the expression of genes associated with translation class, including genes encoding the 30S and 50S ribosomal proteins (the two most downregulated 30S and 50S genes are listed in Table 5) and aminoacyl-tRNA synthetase associated with glutamine (*glnS*), glycine (*glyS*), leucine (*leuS*), lysine (*lysS*), proline (*proS*), valine (*valS*), and aspartate (*aspS*). In addition, the expression of genes involved in the biosynthesis of several amino acids, including histidine (*hisF1* and *hisG*), arginine (*argB*, *argG*, and *argH*), cysteine (c*ysM*), and tryptophan (*trpA* and *trpB*), was also decreased after exposure to *K*_F_.

RNA-seq data showed a downregulation in the expression of genes associated with DNA replication (*dnaJ*, *dnaK*, *dnaA,* and *holC*) and repair mechanism (*mutL*, *phr*, *sbcD*, *uvrC*, *uvrD*, and *recG*) (Table 5).

This group also contained several genes related to cytochrome c, which is highly expressed under microaerobic conditions. These genes with downregulated expression include those encoding elements in cytochrome c (*ccmEG*, PA1600, and PA4571) and cbb3-1 cytochrome c terminal oxidases (*ccoO1Q1* and PA4133).

The expression of NADH dehydrogenase I chain subunits (*nuoD* and *nuoE*) in the oxidative phosphorylation pathway was significantly decreased in *K*_F_-treated samples, with log2-fold changes of −2.216 and −2.162, respectively. Table 5 also shows that the expression of genes involved in energy production in the absence of oxygen through denitrification was decreased after *K*_F_ treatment. These genes with downregulated expression include those encoding nitrate/nitrite transporters (*narK*_1_ and *narK*_2_), the dissimilatory nitrate reductase (*narG* and *narJ*), the two-component regulator NarL, and the transcriptional regulator Dnr.

#### 3.1.6. Group VI: Genes with Downregulated Expression at 6 h and 24 h

This group consisted of genes with downregulated expression at both 6 h and 24 h (Table 6). Growth under *K*_F_ exposure conditions induced the downregulation of the expression of genes encoding heat shock proteins (*hslVU*, *htpG,* and *grpE*). The expression of the *clpB* gene, which encodes the ATP-binding subunit protease, was also downregulated under *K*_F_ exposure conditions.

In this group, we observed the downregulation of the expression of genes involved in the initiation stage of biofilm formation (*bfiR* and *bfiS*) in *K*_F_-treated *P. aeruginosa* samples. Biofilm formation in *P. aeruginosa* is regulated by three novel two-component regulatory systems that are involved in (i) the initiation of biofilm formation (BfiRS), (ii) biofilm maturation (BfmRS), and (iii) microcolony formation (MifRS).

### 3.2. Validation of NGS Results Using Quantitative Real-Time PCR (qRT-PCR)

Four genes identified from RNA-seq data (*uvrD*, *sodM*, *fumC1*, and *rpsL*) were selected for qRT-PCR analysis. *nadB* (PA0761) was chosen as the reference control gene that exhibited no change in our transcriptomic data at two treatment times. qRT-PCR data showed the same trend of either upregulation or downregulation of the genes as that in NGS, thereby validating our NGS results (Table 7). The variations were due to the difference in the sensitivity of the two assays.

## 4. Discussion

Previous studies have elucidated that *K*_F_ can inhibit *P. aeruginosa* growth [6, 7]. In regard to this inhibitory effect, the approach of transcriptomic analysis is useful to identify the differentially expressed genes in this bacterium. The transcriptome profiles of *P. aeruginosa* treated with *K*_F_ were examined to demonstrate the changes in gene expression at two time points (6 h and 24 h incubation). Functional analyses were performed to clarify the possible mechanisms underlying the changes in gene expression from a global perspective. In addition, qRT-PCR was used to confirm the RNA-seq results of select genes.

The type III secretion system (T3SS) regulates the virulence of many pathogenic bacteria [10]. The T3SS system is essential for the export of effector proteins through a needle-like structure directly inside target host cells [10]. Transcriptome data showed the continuous upregulation of all T3SS apparatus, regulators, and effector proteins in *P. aeruginosa* at 6 h and 24 h of *K*_F_ treatment (Table 1). Interestingly, the expression of *P. aeruginosa* genes involved in the flagella assembly pathway, which mediates swimming motility and functions in biofilm development, was increased [11]. These findings indicate that the T3SS system and flagella assembly pathway are tuned by different environmental stresses, which might be an essential survival strategy for this bacterium [12].

As shown in Table 2, gene expression analysis of *P. aeruginosa* grown in *K*_F_ for 6 h displayed an upregulation of the operon *fabAB* (Table 2), which is involved in the biosynthesis of unsaturated fatty acids (UFAs). UFAs are required to maintain the fluidity of bacterial membranes [13]. Thus, we assume that the membrane lipid composition might be altered to allow growth under *K*_F_ exposure conditions.

*Pseudomonas aeruginosa* has a highly complex respiratory chain with multiple terminal oxidases and can respire both oxygen and nitrogen oxides [14, 15]. Under anaerobic conditions, *P. aeruginosa* can respire through denitrification [16]. In this process, four reductases (nitrate-, nitrite-, NO-, and nitrous oxide reductases) allow bacterial growth [17]. Thus, during this process, molecular oxygen is replaced by nitrate as the terminal electron acceptor [18]. The *P. aeruginosa* genome encodes three NADH dehydrogenase chains (NADH-I, NADH-II, and Nqr). When oxygen is not available, the NADH-I chain encoded by the *nuoA-N* operon is required to translocate protons and oxidize NADH to NAD^+^ [19, 20]. Chain I links the NADH ubiquinone electron transfer to the transmembrane transport of protons, leading to the production of a proton motive force that is fundamental for ATP synthesis [21]. In prokaryotic microorganisms, ATP synthesis generally occurs by glycolysis using substrate-level phosphorylation and by the oxidative phosphorylation pathway [11]. In the present study, genes associated with the NADH dehydrogenase I chain, which is involved in the oxidative phosphorylation pathway, displayed a very strong induction at 6 h (Table 2), followed by a reduction at 24 h (Table 5). The expression of chain I subunits (*nuo*-operon) in the oxidative phosphorylation pathway was increased in *K*_F_-treated samples at 6 h. The NADH-I chain is coupled to the denitrification pathway [22, 23]. The upregulation of genes encoding NADH-I chain was paralleled by the increased expression of *anr* gene involved in controlling *P. aeruginosa* gene expression under anaerobic conditions, suggesting that *K*_F_-treated cells underwent a switch to anaerobic respiration in response to oxidative stress. Zimmermann et al. [24] noted that the *anr* deletion mutant of *P. aeruginosa* does not grow anaerobically. In addition, the expression of genes encoding several elements of the ATP-binding cassette transporters (ABCs), which exist in all bacterial species and provide a pathway for substrates to cross the cell membrane [25], was upregulated (Table 1). Interestingly, the growth of *P. aeruginosa* under *K*_F_ exposure conditions at two time points led to the increased expression of genes encoding ABC transporters of amino acids, carbohydrates, and inorganic ions (Table 1). As amino acids are key intermediates in bacterial metabolism, the increase in the ABC transporter proteins led to increased amino acid or peptide uptake. In conclusion, to maintain energy consumption, the cell increases the oxidative phosphorylation pathway and the expression of ATP synthase to produce ATP.

During host infection, *P. aeruginosa* utilizes several systems to acquire iron from the surrounding environment [26]. The iron acquisition mechanisms include the production of siderophores (pyoverdine and pyochelin) and heme uptake [27]. Transcriptomic analysis showed a downregulation of the expression of genes involved in iron acquisition in *K*_F_-treated samples at 24 h of *P. aeruginosa* growth. As shown in Table 5, TonB-dependent siderophore receptor (*tonB*) and haemeoxygenase (*hemO*) showed a reduction in the expression level at 24 h. The downregulation of the expression of *pchR*-encoding elements involved in iron Fe^3+^ acquisition was also observed. In addition, the expression of genes highly regulated by iron starvation was repressed by *K*_F_ treatment. These genes encode members of the ECF subfamily, which is mainly associated with extracellular functions that include the regulation of periplasmic stress, iron transport, metal ion efflux systems, alginate secretion, and synthesis of membrane-localized carotenoids [28]. Consequently, the results suggested that *K*_F_-treated cells underwent conditions of excess intracellular iron, which led to the downregulation of the expression of genes regulated by the ferric uptake regulator (Fur) required for iron acquisition. Ochsner et al. [29] reported that the Fur protein uses Fe^2+^ as a cofactor and binds to Fur-Fe^2+^, resulting in the repression of the genes encoding pyochelin and pyoverdin proteins in iron-replete environments. Furthermore, transcriptomic analysis also showed the downregulation of the expression of genes coding for the components of the DNA replication and repair machinery in *P. aeruginosa* at 24 h of *K*_F_ treatment. A superoxide (O_2_^−^) byproduct is formed by the autoxidation of a variety of reduced electron carriers and redox enzymes [30]. O_2_^−^ is implicated in the production of oxidative DNA damage by the steady release of iron from storage proteins into the cytosol, and thus, the free iron binds DNA and catalyses electron transfer from the reductant to H_2_O_2_ [31, 32]. The resultant ferryl or hydroxyl radical attacks the adjacent DNA [33]. The repression of genes encoding DNA repair proteins was coupled with the repression of genes involved in iron regulation at 24 h, suggesting that *K*_F_-treated cells were exposed to an excess concentration of intracellular free iron, leading to either hydroxyl or ferryl radical production, which promotes oxidative DNA damage by increasing the amount of DNA-bound iron. Oxidative DNA damage was also evident by the downregulation of the expression of genes involved in defence (*sodM*) against reactive oxygen species.

*P. aeruginosa* pathogenicity depends on the production and secretion of a large variety of virulence factors, including pyocin S2, in response to host environments. pyocin S2 is a protease-sensitive bacteriocin produced by *P. aeruginosa* that kills sensitive cells by damaging chromosomal DNA through its DNase activity and the inhibition of lipid synthesis [34]. RNA-seq analysis showed reduced expression levels of pyocin S2 protease at 6 h of exposure to *K*_F_ (Table 3). As shown in Table 5, the growth of *P. aeruginosa* under *K* treatment conditions at 24 h led to the decreased expression of genes involved in the LPS biosynthesis process (*lpxA*, *lpxB*, *waaF*, *waaG*, *waaP*, PA4998, PA5007, PA5008, and *rmlA*). LPS is the major component defining the outer membrane of Gram-negative bacteria. The outer membrane is essential for viability and mediates virulence and resistance to toxic and antibacterial agents [35]. Interestingly, a previous study revealed that the *waaP* gene in *P. aeruginosa* is required to produce full-length LPS, which is recognized by the outer membrane transport assembly machinery in this bacterium [36]. Therefore, *waaP* may constitute a good target for the development of novel antipseudomonal agents. Our previous observation is consistent with this finding. Transmission electron microscopy studies have revealed that cells treated with *K*_F_ exhibit severe membrane damage concurrent with the disruption of membrane integrity, leading to the loss of intracellular material at 24 h of incubation [7]. These results suggest that LPS biosynthesis may be inhibited at 24 h *K*_F_ exposure. In addition, the MexGHI-OpmD efflux pump system has been implicated in the efflux of xenobiotics, including the antibiotic norfloxacin and the heterocyclic dye acriflavine [37], and the transport of phenazine molecules [38]. Interestingly, the downregulation of the MexGHI-OpmD system observed in the RNA-seq data was coupled with a reduction in the phenazine biosynthesis process *K*_F_ at 24 h of *P. aeruginosa* treated with *K*_F_ (Table 5). The opportunistic pathogen *P. aeruginosa* is well known for its production of bright blue phenazine pyocyanin, which contributes to the colouration of sputum and pus associated with infections and interferes with multiple host cellular functions [39]. In response to *K*_F_ treatment, *P. aeruginosa* repressed the expression of the secretory machinery (Sec system), responsible for the secretion of virulence factors, extracellular degradative enzymes, and other toxins, enabling adaptation to a wide range of ecological niches [40]. Therefore, taken together, these data reveal the marked remodelling of gene transcription characterized by an early and late reduction in the expression of several genes associated with virulence factors of *P. aeruginosa* in response to *K*_F_ treatment.

Bacteria can form biofilms on living or nonliving surfaces and can be prevalent in natural, industrial, and hospital settings. Bacterial motility and adhesion are critical for biofilm development [41]. The type IV pili in *P. aeruginosa* play an important role in the adherence to epithelial cells and microbial intra and interspecies competition, while flagella filament-mediated motility enables bacteria to reach a surface and then divide and spread along the surface [42]. In response to *K*_F_ treatment at 24 h, the downregulation of type IV and flagellin type B (*fliC*) genes observed in RNA-seq (Table 5) was paralleled by the decreased expression of genes involved in biofilm formation in *P. aeruginosa* (Table 6). These findings may indicate that *K*_F_ treatment affects genes involved in biofilm formation and motility. As a consequence of these combined factors, we thus hypothesise that the swimming and biofilm formation ability of *P. aeruginosa* would be inhibited under *K*_F_ treatment conditions.

The treatment of *P. aeruginosa* with *K*_F_ for 24 h led to the decreased expression of genes encoding the aminoacyl-tRNA synthetases glutamine, glycine, leucine, lysine, proline, valine, and aspartate. Furthermore, genes associated with the biosynthesis of several amino acids, including histidine, arginine, cysteine, and tryptophan, were also expressed at reduced levels in *K*_F_-treated samples at 24 h (Table 5). RNA-seq data showed that the downregulation of the expression of genes *hslVU*, *htpG*, and *grpE* involved in the degradation of unfolded or misfolded proteins that accumulate in the periplasm [43], following heat shock or other stress conditions was coupled with the decreased expression of the *clpB* gene encoding an ATP-dependent protease, which functions as part of the chaperone network essential for the recovery of stress-induced protein aggregates [44] (Table 6). The altered expression of these genes at two *K*_F_ exposure time points may be indicative of their essential function in cellular responses to environmental stress. As a consequence of these combined factors, we thus assume that protein synthesis in *P. aeruginosa* might be affected by *K*_F_ treatment.

## 5. Conclusion

The crisis of the antibiotic resistance demands to be met with concerted efforts across many disciplines and areas of expertise. Natural products are mainstays of drugs and still play an essential role in providing chemical diversity, despite a reduced interest shown by pharmaceutical companies. Herein, we could prove efficacy of *K*_F_ against one of the most notorious pathogen *P. aeruginosa*. The *K*_F_ compound is more likely to have multitargets inside the test *P. aeruginosa*. To the best of our knowledge, the current study is the first report describing the antibacterial effect of *K*_F_ on *P. aeruginosa* at the gene expression level through transcriptomic analysis, revealing the regulation of various genes involved in cellular processes that lead to the destabilization of this bacterium. The transcriptomic analysis showed that *K*_F_ increases the expression of genes involved in the electron transport chain (NADH-I), resulting in the induction of ATP synthesis. *K*_F_ also increased the expression of genes associated with ATP-binding cassette transporters, flagella, type III secretion system proteins, and DNA replication and repair, which may further affect nutrient uptake, motility, and growth. The major mechanisms through which *K*_F_ seems to exert its antibacterial effect on *P. aeruginosa* are by the repression of a broad range of virulence factors associated with LPS biosynthesis, iron homeostasis, cytotoxic pigment pyocyanin production, and motility and adhesion that are representative of an acute *P. aeruginosa* infection profile. Taken together, the present study is a good demonstration of the therapeutic usefulness of the natural product from plant in validating the traditional medicine, i.e., *M. malabathricum*, very common in Malaysia. Specifically, attenuations of bacterial virulence factors are likely to be effective solutions in this therapeutic area. Although the current study offers a possible regulatory network of *P. aeruginosa* induced by *K*_F_ treatment, further studies will focus on the protein level expression of the target genes. In general, this study has generated scientific evidence that natural product research is perfectly positioned to address and solve the present bacterial resistance crisis and the closely linked antibiotic discovery gap.

## Figures and Tables

**Table 1:** List of the group I genes with upregulated expression at 6 h and 24 h.

Genes	6 h fold change	*p* value	24 h fold change	*p* value	Description	Functional class
*agtA*	20.45	0	9.21	0	ABC-type spermidine/putrescine transport systems, ATPase components	TSMs
*agtB*	32.34	0	8.09	0	ABC-spermidine/putrescine-binding periplasmic protein	TSMs
*agtC*	25.25	0	4.57	0	ABC-type spermidine/putrescine transport system, permease component I	TSMs; MPs
*agtD*	15.20	1.68*E* − 03	3.00	2.68*E* − 07	ABC-type spermidine/putrescine transport system, permease component II	TSMs; MPs
PA4500	8.02	0	3.11	0	ABC-type dipeptide transport system, periplasmic component	TSMs
PA3190	7.60	2.64*E* − 04	2.09	0	ABC-type sugar transport system, periplasmic component	TSMs
PA3514	6.95	6.67*E* − 04	5.08	0	ABC-type nitrate/sulfonate/bicarbonate transport system, ATPase component	TSMs
*spcS*	35.38	0	3.94	0	Specific *Pseudomonas* chaperone for ExoS, SpcS	SFs; PSEA
*pcrV*	14.54	0	5.89	0	Type III protein secretion system complex	PSEA
*pcrH*	7.52	0	4.94	0	Regulatory protein PcrH	SFs; PSEA
*popB*	11.31	0	4.79	0	Translocator protein PopB	PSEA
*popD*	9.67	0	3.48	0	Translocator outer membrane protein PopD precursor	PSEA
*exsC*	10.98	0	4.09	0	ExsC, exoenzyme S synthesis protein C precursor	PSEA
*exsE*	8.61	2.32*E* − 06	4.62	0	ExsE	PSEA
*exsD*	11.895	0	4.572	0	ExsD	PSEA
*pscG*	7.60	4.15*E* − 03	2.31	6.10*E* − 03	Type III export protein PscG	PSEA; CHSPs
*exoS*	13.43	0	6.71	0	Exoenzyme S	SFs
*exoT*	9.36	0	4.50	0	Exoenzyme T	SFs
*exoY*	31.08	0	5.82	0	Adenylate cyclase ExoY	SFs

**Table 2:** List of the group II genes with upregulated expression at 6 h.

Genes	6 h (fold change)	*p* value	Description	Functional class
*hisC1*	4.814	0.013	Histidinol-phosphate aminotransferase	AABM
*HisE*	3.111	4.06*E* − 08	Phosphoribosyl-ATP pyrophosphohydrolase	AABM
*ArgF*	2.723	2.16*E* − 03	Ornithine carbamoyltransferase, anabolic	AABM
*ArgJ*	2.601	9.75*E* − 03	Glutamate N-acetyltransferase	AABM
*ilvA1*	2.117	0.021	Threonine dehydratase, biosynthetic	AABM
*LeuA*	5.244	0	2-Isopropylmalate synthase	AABM
*LeuC*	2.387	7.87*E* − 12	3-Isopropylmalate dehydratase large subunit	AABM
*PheA*	2.1	0.018	Chorismate mutase	AABM
*RpsL*	2.56	0	30S ribosomal protein S12	TPTMD
*RplA*	3.679	0	50S ribosomal protein L1	TPTMD
*TrpS*	3.501	1.62*E* − 05	Tryptophanyl-tRNA synthetase	TPTMD; AABM
*TyrZ*	3.239	0	Tyrosyl-tRNA synthetase 2	TPTMD; AABM
*GlyQ*	2.431	0	Glycyl-tRNA synthetase alpha chain	TPTMD; AABM
*GlnE*	2.912	3.52*E* − 07	Glutamine synthetase adenylyltransferase	TPTMD
*ValS*	2.356	3.57*E* − 10	Valyl-tRNA synthetase	TPTMD; AABM
*ProS*	2.274	2.36*E* − 09	Prolyl-tRNA synthetase	TPTMD; AABM
*CysS*	2.152	2.30*E* − 05	Cysteinyl-tRNA synthetase	TPTMD; AABM
*IleS*	2.02	1.72*E* − 08	Isoleucyl-tRNA synthetase	TPTMD; AABM
*InfC*	7.182	0	Translation initiation factor IF-3	TPTMD
*fusA2*	2.313	5.73*E* − 07	Elongation factor G	TPTMD
*PrfC*	3.564	0.013	Peptide chain release factor 3	TPTMD
PA5195	3.589	0.028	Probable heat shock protein	CHSPs
*HscB*	3.187	2.35*E* − 05	Heat shock protein HscB	CHSPs
*AccB*	6.303	0	Biotin carboxyl carrier protein (BCCP)	FAPM
*AccD*	3.579	6.23*E* − 12	Acetyl-CoA carboxylase beta subunit	FAPM
*FabA*	2.258	2.67*E* − 06	Beta-hydroxydecanoyl-ACP dehydrase	FAPM
*FabB*	2.96	0	Beta-ketoacyl-ACP synthase I	FAPM
*NorB*	8.614	1.58*E* − 03	Nitric oxide reductase subunit B	EM
*Anr*	3.84	0	Transcriptional regulator Anr	EM
*NuoB*	4.162	0	NADH dehydrogenase I chain B	EM
*NuoD*	2.982	0	NADH dehydrogenase I chain C,D	EM
*NuoF*	2.657	5.14*E* − 09	NADH dehydrogenase I chain F	EM
*NuoG*	2.761	0	NADH dehydrogenase I chain G	EM
*NuoH*	3.026	9.62*E* − 06	NADH dehydrogenase I chain H	EM
*NuoI*	7.185	3.21*E* − 14	NADH dehydrogenase I chain I	EM
*NuoJ*	4.172	3.27*E* − 03	NADH dehydrogenase I chain J	EM
*NuoL*	3.459	0	NADH dehydrogenase I chain L	EM
*NuoM*	2.884	5.40*E* − 04	NADH dehydrogenase I chain M	EM
*NuoN*	5.008	2.12*E* − 07	NADH dehydrogenase I chain N	EM
*FlgB*	5.197	0	Flagellar basal body rod protein FlgB	CWLC; MA
*FlgC*	2.907	8.96*E* − 10	Flagellar basal body rod protein FlgC	CWLC; MA
*FlgD*	4.125	4.38*E* − 14	Flagellar basal body rod modification protein FlgD	CWLC; MA
*FlgE*	4.984	0	Flagellar hook protein FlgE	CWLC; MA
*FlgF*	4.623	2.03*E* − 14	Flagellar basal body rod protein FlgF	CWLC; MA
*FlgG*	3.297	0	Flagellar basal body rod protein FlgG	CWLC; MA
*FlgI*	2.4	0.01	Flagellar P-ring protein precursor FlgI	CWLC; MA
*FlgJ*	4.057	3.23*E* − 13	Flagellar protein FlgJ	CWLC; MA
*FlgK*	3.85	2.24*E* − 10	Flagellar hook-associated protein 1 FlgK	CWLC; MA
*FliE*	4.534	1.18*E* − 11	Flagellar hook-basal body complex protein FliE	CWLC; MA
*FliF*	3.433	0	Flagella M-ring outer membrane protein precursor	CWLC; MA
*FliG*	2.635	6.34*E* − 10	Flagellar motor switch protein FliG	CWLC; MA

**Table 3:** List of the group III genes with downregulated expression at 6 h.

Genes	6 h (fold change)	24 h (fold change)	Description	Functional class
*Pys2*	−4.084	0	Pyocin S2	AP; SFs
*imm2*	−2.56	0	Pyocin S2 immunity protein	AP
*cobO*	−2.684	8.27*E* − 04	Cob (I) alamin adenosyltransferase	BCPGCs
*cobD*	−4.229	0.012	Cobalamin biosynthetic protein CobD	BCPGCs
*cobU*	−4.306	7.36*E* − 04	Nicotinate-nucleotide-dimethylbenzimidazole phosphoribosyltransferase	BCPGCs
*cobJ*	−4.9	0	Precorrin-3 methylase CobJ	BCPGCs

**Table 4:** List of the group IV genes with upregulated expression at 24 h.

Genes	24 h (fold change)	*p* value	Description	Functional class
*dctP*	2.879	0	DctP	MPs; TSMs
*dctQ*	2.419	1.04*E* − 06	DctQ	MPs; TSMs
*dctM*	2.206	4.35E − 05	DctM	MPs; TSMs

**Table 5:** List of the group V genes with downregulated expression at 24 h.

Genes	24 h (fold change)	*p* value	Description	Functional class
*pchR*	−3.116	2.23*E* − 15	Transcriptional regulator PchR	TRs
PA0471	−2.863	1.61*E* − 05	Fe^2+^-dicitrate sensor, membrane component	TCRSs; MPs; TRs
*fiuI*	−2.171	1.62*E* − 03	Fe^2+^-dicitrate sensor, membrane component	TRs
PA1300	−2.201	5.72*E* − 09	Sigma-70 factor, ECF subfamily	TRs
PA1301	2.386	0.014	Probable transmembrane sensor	MPs; TRs
PA3899	−3.481	0	Probable sigma-70 factor, ECF subfamily	TRs
PA3900	−2.505	0.019	Fe^2+^-dicitrate sensor, membrane component	MPs; TRs
PA4895	−5.965	1.26*E* − 09	Fe^2+^-dicitrate sensor, membrane component	MPs; TRs
PA4896	−3.644	8.33*E* − 11	Sigma-70 factor, ECF subfamily	TRs
PA0149	−3.859	1.15*E* − 08	Probable sigma-70 factor, ECF subfamily	TRs
*femI*	−3.628	0	ECF sigma factor, FemI	TRs
PA2896	−2.495	0	Probable sigma-70 factor, ECF subfamily	TRs
*tonB1*	−2.067	0	Periplasmic protein TonB, links inner and outer membranes	TSMs
PA4156	−11.36	0	Probable TonB-dependent receptor	TSMs
*fumC1*	−4.599	0	Fumarate hydratase	EM
*sodM*	−3.955	0	Superoxide dismutase	AP
*hemO*	−3.581	0	Heme oxygenase	BCPGCs
PA0853	−3.242	2.66*E* − 13	Oxidoreductase	PEs
PA3768	−2.622	0	Probable metallo-oxidoreductase	PEs
*phzA1*	−4.295	1.53*E* − 07	Probable phenazine biosynthesis protein	SFs
*phzB1*	−4.708	−4.708	Probable phenazine biosynthesis protein	SFs
*phzC1*	−2.577	−2.577	Phenazine biosynthesis protein PhzC	SFs
*phzA2*	−2.625	−2.625	Probable phenazine biosynthesis protein	SFs
*phzB2*	−3.848	−3.848	Probable phenazine biosynthesis protein	SFs
*phzM*	−2.125	1.14*E* − 10	Probable phenazine-specific methyltransferase	PEs
*phzS*	−2.241	0	Flavin-containing monooxygenase	PEs
*secA*	−2.166	0	Secretion protein SecA	PSEA
*secB*	−2.579	0	Secretion protein SecB	PSEA
*secD*	−3.234	0	Secretion protein SecD	PSEA; MPs
*mexG*	−2.119	7.29*E* − 03	Membrane protein	MPs
*mexH*	−9.088	0	Probable resistance-nodulation-cell division (RND) efflux membrane fusion protein precursor	TSMs
*mexI*	−5.758	0	Probable resistance-nodulation-cell division (RND) efflux transporter	TSMs; MPs
*opmD*	−3.241	0	Outer membrane protein precursor	TSMs; MPs
*lpxB*	−2.148	7.24*E* − 03	Lipid A-disaccharide synthase	CWLC
*lpxA*	−2.274	0	UDP-N-acetylglucosamine acyltransferase	CWLC
*waaP*	−3.174	2.75*E* − 14	Lipopolysaccharide kinase WaaP	CWLC
*waaG*	−2.437	0	UDP-glucose:(heptosyl) LPS alpha 1,3-glucosyltransferase WaaG	CWLC
*waaF*	−2.283	1.48*E* − 08	Heptosyltransferase II	CWLC
PA4998	−2.482	6.80*E* − 12	Aminoglycoside 3′-phosphotransferase (APH) and choline kinase family	CWLC
PA5007	−3.124	4.80*E* − 08	Mn^2+−^dependent serine/threonine protein kinase	PEs
PA5008	−3.187	7.22*E* − 15	RIO-like serine/threonine protein kinase fused to N-terminal HTH domain	PEs
*rmlA*	−2.554	0	Glucose-1-phosphate thymidylyltransferase	CWLC
*pilD*	−2.377	0	Type 4 prepilin peptidase PilD	SFs; PSEA; MA
*pilF*	−2.053	0	Type 4 fimbrial biogenesis protein PilF	PSEA; MA
*pilM*	−3.074	0	Type 4 fimbrial biogenesis protein PilM	MA
*pilN*	−3.871	0	Type 4 fimbrial biogenesis protein PilN	MA
*pilO*	−4.476	0	Type 4 fimbrial biogenesis protein PilO	MA
*pilP*	−3.956	0	Type 4 fimbrial biogenesis protein PilP	MA
*pilQ*	−3.219	0	Type 4 fimbrial biogenesis outer membrane protein PilQ precursor	MA
*pilU*	−2.226	0	Twitching motility protein PilU	MA
*pilV*	−2.382	0	Type 4 fimbrial biogenesis protein PilV	MA
*pilW*	−2.516	0	Type 4 fimbrial biogenesis protein PilW	MA
*pilX*	−2.366	0	Type 4 fimbrial biogenesis protein PilX	MA
*pilY1*	−2.025	0	Type 4 fimbrial biogenesis protein PilY1	MA
*pilG*	−2.713	0	Twitching motility protein PilG	TCRSs; MA; CT
*pilH*	−3.112	0	Twitching motility protein PilH	TCRSs; MA; CT
*pilI*	−2.731	2.20*E* − 09	Twitching motility protein PilI	MA; CT
*pilJ*	−5.282	0	Twitching motility protein PilJ	MA; CT
*Vfr*	−2.047	0	Transcriptional regulator vfr	TRs
*chpA*	−2.124	0	Component of chemotactic signal transduction system	TCRSs; MA; CT
*chpB*	−2.315	1.15*E* − 05	Probable methylesterase	CT
*fliC*	−2.145	0	Flagellin type B	MA
*rpsK*	−2.592	0	30S ribosomal protein S11	TPTMD
*rplA*	−2.375	0	50S ribosomal protein L1	TPTMD
*glnS*	−2.102	0	Glutaminyl-tRNA synthetase	TPTMD; AABM
*glyS*	−2.162	2.11*E* − 13	Glycyl-tRNA synthetase beta chain	TPTMD; AABM
*leuS*	−2.131	0	Leucyl-tRNA synthetase	TPTMD; AABM
*lysS*	−2.38	0	Lysyl-tRNA synthetase	TPTMD; AABM
*proS*	−2.764	0	Prolyl-tRNA synthetase	TPTMD; AABM
*valS*	−2.13	0	Valyl-tRNA synthetase	TPTMD; AABM
*aspS*	−2.129	0	Aspartyl-tRNA synthetase	T-RNA-PD; TPTMD
*hisF1*	−3.599	1.02*E* − 11	Imidazole glycerol-phosphate synthase, cyclase subunit	AABM
*hisG*	−2.863	2.04*E* − 10	ATP-phosphoribosyltransferase	AABM
*argB*	−2.436	0	Acetylglutamate kinase	AABM
*argG*	−2.428	0	Argininosuccinate synthase	AABM
*argH*	−2.127	0	Argininosuccinate lyase	AABM
*cysM*	−3.436	0	Cysteine synthase B	AABM
*trpA*	−4.405	0	Tryptophan synthase alpha chain	AABM
*trpB*	−6.527	0	Tryptophan synthase beta chain	AABM
*hslU*	−4.255	0	Heat shock protein HslU	CHSPs
*hslV*	−3.742	0	Heat shock protein HslV	CHSPs
*htpG*	−2.835	0	Heat shock protein HtpG	CHSPs
*htpX*	−2.557	0	Heat shock protein HtpX	AP
*dnaA*	−2.631	0	Chromosomal replication initiation protein	DNA-RRMR
*dnaJ*	−2.528	0	DnaJ protein	DNA-RRMR; CHSPs; AP
*dnaK*	−3.087	0	DnaK protein	DNA-RRMR; CHSPs; AP
*holC*	−2.701	5.25*E* − 09	DNA polymerase III, chi subunit	DNA-RRMR
*mutL*	−2.563	0	DNA mismatch repair protein MutL	DNA-RRMR
*Phr*	−3.012	2.14*E* − 12	Deoxyribodipyrimidine photolyase	DNA-RRMR
*sbcD*	−2.056	2.40*E* − 13	Exonuclease SbcD	DNA-RRMR
*recG*	−2.105	7.52*E* − 16	ATP-dependent DNA helicase RecG	DNA-RRMR; TRs
*uvrC*	−2.622	0	Excinuclease ABC subunit C	DNA-RRMR
*uvrD*	−3.412	0	DNA helicase II	DNA-RRMR
*ccmE*	−2.297	7.41*E* − 10	Cytochrome C-type biogenesis protein CcmE	EM
*ccmG*	−2.001	3.24*E* − 06	Cytochrome C biogenesis protein CcmG	TPTMD; CHSPs; EM
PA1600	−2.819	2.31*E* − 06	Probable cytochrome c	EM
PA4571	−2.708	0	Probable cytochrome c	EM
PA4133	−5.344	0	Cytochrome c oxidase subunit (cbb3-type)	EM
*ccoO1*	−2.475	0	Cytochrome c oxidase, cbb3-type, CcoO subunit	EM
*ccoQ1*	−2.235	1.16*E* − 04	Cytochrome c oxidase, cbb3-type, CcoQ subunit	EM
*nuoD*	−2.216	0	NADH dehydrogenase I chain C,D	EM
*nuoE*	−2.162	9.15*E* − 13	NADH dehydrogenase I chain E	EM
*narK1*	−2.541	2.23*E* − 15	Nitrite extrusion protein 1	MPs; TSMs
*narK2*	−5.637	0	Nitrite extrusion protein 2	MPs; TSMs
*narG*	−4.157	0	Respiratory nitrate reductase alpha chain	EM
*narJ*	−2.386	0.014	Respiratory nitrate reductase delta chain	EM
*narL*	−2.09	0	Two-component response regulator NarL	EM; TCRSs
*Dnr*	−2.065	8.03*E* − 14	Transcriptional regulator Dnr	TRs

**Table 6:** List of the group VI genes with downregulated expression at 6 h and 24 h.

Genes	6 h (fold change)	*p* value	24 h (fold change)	*p* value	Description	Functional class
*htpG*	− 4.483	0	− 2.835	0	Heat shock protein HtpG	CHSPs
*HslV*	− 5.567	0	− 3.742	0	Heat shock protein HslV	CHSPs
*HslU*	− 3.379	0	− 4.255	0	Heat shock protein HslU	CHSPs
*grpE*	− 2.265	0	− 3.718	0	Heat shock protein GrpE	DNA-RRMR; CHSPs
*ClpB*	− 5.018	0	− 2.312	0	ATP-binding subunits of clp protease and DnaK/DnaJ chaperones	TPTMD
*BfiR*	− 2.707	0.033	− 2.584	0	Response regulator	TRs; TCRSs
*BfiS*	− 4.934	6.31*E* − 15	− 2.714	0	Signal transduction histidine kinase regulating C4-dicarboxylate transport system	TCRSs

**Table 7:** Transcript level comparison of *P. aeruginosa* genes between qRT-PCR and NGS. qRT-PCR is the mean of two biological replicates with three technical replicates for each gene. Reference gene (*nadB*): L-aspartate oxidase, *uvrD*, and *sodM* were downregulated at 24 h with no change at 6 h; *fumC1* was upregulated at 6 h and downregulated at 24 h; *rpsL* was upregulated at 6 h with NC at 24 h exposure.

Genes ID	Gene symbol	NGS		qRT-PCR		Primers	Length (bp)	Description
		Fold change		Fold change				
		6 h	24 h	6 h	24 h	5′-sequence-3′		
PA5443	*uvrD*	NC	−3.412 ± 0	NC	−2.54 ± 0.01	GTGCGCCTGTCCAATAC	17	DNA helicase II
						GCCTTCGAAGTTGAGGATAG	20	
PA4468	*sodM*	NC	−3.955 ± 0	NC	−2.44 ± 0.01	GAGCAGCCGGTGGAAAGTCT	20	Superoxide dismutase
						GCGACATCACGGTCCAGAAC	20	
PA4470	*fumC1*	3.618 ± 0	−4.599 ± 0	3.48 ± 0.69	−5.39 ± 0	TCGGGCAACTTCGAACTGAA	20	Fumarate hydratase
						GAGCTTGCCCTGGTTGACCT	20	
PA4268	*rpsL*	2.56 ± 0	NC	3.53 ± 0.53	NC	CGGCACTGCGTAAGGTATGC	20	30S ribosomal protein S12
						CCCGGAAGGTCCTTTACACG	20	
PA0761^*∗*^	*nadB*		Reference gene			CTACCTTTATACCAGCAATCCC	22	L-aspartate oxidase
						CGGTGATGAGGAAACTCTTG	20	

## Data Availability

The data used to support the findings of this study are included within the supplementary information files.

## Abbreviations

AP: Adaptation, protection
SFs: Secreted factors (toxins, enzymes, and alginate)
AABM: Amino acid biosynthesis and metabolism
T-RNA-PD: Transcription, RNA processing, and degradation
BCPGCs: Biosynthesis of cofactors, prosthetic groups, and carriers
TRs: Transcriptional regulators
CWLC: Cell wall/LPS/capsule
TPTMD: Translation, post-translational modification, degradation
CHSPs: Chaperones and heat shock proteins
TSMs: Transport of small molecules
CT: Chemotaxis
TCRSs: Two-component regulatory systems
DNA-RRMR: DNA replication, recombination, modification, and repair
HUU: Hypothetical, unclassified, and unknown
EM: Energy metabolism
PSEA: Protein secretion/export apparatus
FAPM: Fatty acid and phospholipid metabolism
PEs: Putative enzymes
MA: Motility and attachment
MPs: Membrane proteins.
